# Children’s representations of school support for HIV-affected peers in rural Zimbabwe

**DOI:** 10.1186/1471-2458-14-402

**Published:** 2014-04-26

**Authors:** Catherine Campbell, Louise Andersen, Alice Mutsikiwa, Claudius Madanhire, Morten Skovdal, Constance Nyamukapa, Simon Gregson

**Affiliations:** 1 Department of Social Psychology, The London School of Economics and Political Science, Houghton Street, London, WC2A 2AE, UK; 2 Department of Public Health, University of Copenhagen, Copenhagen, Denmark; 3 Biomedical Research and Training Institute, Harare, Zimbabwe; 4 Department of Infectious Disease Epidemiology, Imperial College School of Public Health, London, UK; 5 School of Applied Human Sciences, University of KwaZulu Natal, Durban, South Africa

**Keywords:** Children, HIV/AIDS, Policy, Teachers, Peers, Care, Support, Stigma, Social exclusion, Coping, Schools

## Abstract

**Background:**

HIV has left many African children caring for sick relatives, orphaned or themselves HIV-positive, often facing immense challenges in the absence of significant support from adults. With reductions in development funding, public sector budgetary constraints, and a growing emphasis on the importance of indigenous resources in the HIV response, international policy allocates schools a key role in ‘substituting for families’ (Ansell, 2008) in supporting child health and well-being. We explore children’s own accounts of the challenges facing their HIV-affected peers and the role of schools in providing such support.

**Methods:**

Contextualised within a multi-method study of school support for HIV-affected children in rural Zimbabwe, and regarding children’s views as a key resource for child-relevant intervention and policy, 128 school children (10–14) wrote a story about an HIV-affected peer and how school assisted them in tackling their problems.

**Results:**

Children presented harrowing accounts of negative impacts of HIV on the social, physical and mental well-being of peers, and how these manifested in the school setting. Whilst relationships with fellow learners and teachers were said to provide a degree of support, this was patchy and minimal, generally limited to small-scale and often one-off acts of material help or kindness (e.g. teachers giving children pens and exercise books or peers sharing school lunches), with little potential to impact significantly on the wider social drivers of children’s daily challenges. Despite having respect for the enormity of the challenges many HIV-affected peers were coping with, children tended to keep a distance from them. School was depicted as a source of the very bullying, stigma and social exclusion that undermined children’s opportunities for well-being in their lives more generally.

**Conclusions:**

Our findings challenge glib assumptions that schools can serve as a significant ‘indigenous’ supports of the health and well-being of HIV-affected children in the absence of a very significant increase in outside training, support and additional resources. Schools are an extension of communities, with members of school communities subject to many of the same deprivations, anxieties and prejudices that drive the health-limiting exclusion, impoverishment and stigmatisation of HIV-affected children in their households and wider communities.

## Background

What role can schools play in supporting the health and well-being of HIV affected children in Sub-Saharan Africa? Increasingly, international policy-makers [1,2] are pointing to schools as possible “substitutes for families” [3] p. 802 in contexts where HIV/AIDS has disrupted the ability of adults to support children’s physical and emotional health and well-being. We present research conducted in Zimbabwe where 15% of adults aged 15–49 are HIV positive, an age group highly represented amongst parents and relatives of school learners [4]. Around 17% of children have lost one or both parents to the epidemic [5]. In addition around 2.5% of children under 14 are themselves positive. [5]. Child ART coverage is now 46.1% [6], though many challenges stand in the way of optimal treatment adherence by children [7]. Many school learners are affected by HIV/AIDS through having to care for sick or dying parents, being HIV-infected themselves or being orphaned – living in child-headed households or taken in by varyingly supportive relatives or carers [8,9].

We report on a qualitative study nested within a wider multi-method investigation of the potential for schools to support the health and well-being of HIV-affected children in Sub-Saharan Africa. Informed by the classic WHO conceptualisation of health in terms of physical, psychological and social resources for living [10] we have gathered information from the perspectives of children, adults, teachers and community members in rural Zimbabwe. Given the relative neglect of children’s own perspectives in the research literature in this field [11] and the key role that lay people’s own understandings play in responding to health challenges [12], we focus specifically on children’s views of the potential for the school to support their well-being. We draw on stories written by 10 to 14 year old school children, who were invited to draw a picture and write a story about an HIV-affected child in their school.

### Conceptual framework

Our analysis was underpinned by the methodological assumption that, in constructing stories, children draw on the social representations that they and their peers have collectively constructed to give meaning to their social worlds [13,14]. Such social representations constitute the symbolic resources children use to make sense of the everyday and to act within it [15,16]. The importance of including children’s perspectives in research about their well-being has long been emphasised by the tradition of the ‘new social studies of childhood’ [17]. Children’s own views are potentially important resources for public health specialists since they constitute the lens through which children will interpret and respond to efforts to enhance their health and well-being in challenging settings.

In the context of HIV/AIDS management, children’s own views of HIV/AIDS and its impacts have added importance in relation to efforts to combat child-on-child stigma, which is one of the most significant obstacles to effective diagnosis and treatment of HIV/AIDS in this group [13,18]. We pay particular attention to children’s own understandings of the opportunities that schools provide for children to engage in social relationships that enhance opportunities for well-being. Here, our starting assumption is that opportunities for positive social participation increase the likelihood that people will behave in ways that enhance their health [19]. We draw on critical conceptualisations of ‘social capital’ in developing this point.

Extensive work has been done on the role of social capital as a springboard for the development of ‘HIV/AIDS competence’ understood as the likelihood that community members will work collectively to support each other in responding to HIV/AIDS [20]. We understand social capital as the individual and community-level advantages arising from participation in local community networks. We draw on Bourdieu’s [21] view of social capital as those benefits that arise from a person’s engagement in ‘networks of social acquaintances and recognition', which potentially assist an individual in coping with life’s challenges and advancing their interests. Unequal distribution of social capital is a key factor in advancing social inequalities and their associated negative impacts on health [22-24]. Whilst social group memberships may often yield benefits for health and well-being, they can also be a source of health-damaging negative social norms, social exclusion and discrimination against out-group members (‘anti-social capital’) [25,26]. Particular social networks are seldom uniformly good or bad, and we will be alert to children’s views of the ways in which schools potentially both support and undermine their opportunities for health and well-being.

### Empirical literature review

Elsewhere we have reviewed academic research on school support for HIV-affected children in Zimbabwe [Campbell C, Skovdal M, Andersen L: Can schools support children in extreme adversity? A Review. Submitted]. The bulk of this literature focuses on collaborations between schools and outside agencies who bring skills and funding, with a heavy emphasis on programmes training teachers to provide psycho-social support to children. However there is less emphasis on indigenous responses by children and teachers out of the context of externally imposed interventions in contexts where external support is limited for one reason or another, as is the case in Zimbabwe.

The bulk of research in the social capital framework that investigates indigenous community responses to HIV/AIDS in sub-Saharan Africa focuses on local community organisations (women’s groups, faith-based organisations, AIDS support groups and so on, and on NGOs) [27-31]. We expand on this by considering local schools as sources of indigenous social capital [32].

Anecdotal evidence suggests Zimbabwean schools and teachers may be supporting children in all sorts of informal ways, but there is little formal research into this situation. Given that policy emphasis increasingly positions schools as a pillar of the child-related HIV/AIDS response [1,2], there is a need for more systematic attention to existing indigenous responses, and for the development of an evidence base regarding existing indigenous schools-related ‘best practice’. Given current realities of cuts in international development assistance budgets [33], local people and local institutions are increasingly having to respond to problems without a great deal of external help or support.

### The Zimbabwean context

Zimbabwe forms a particularly useful context to explore indigenous responses to HIV/AIDS. In recent years, schools in Zimbabwe have been severely disrupted by political and economic instability and the retreat of many NGOs [7,34]. In 2008 at the height of the economic crisis, salaries paid to teachers were worthless due to high inflation in the country, with many schools ceasing to function for several months. This situation has improved since the collapse of the Zimbabwe dollar and its replacement with the multi-currency system, however teachers remain extremely demoralised, their salaries remain low, and their social status has rapidly declined, leading Shizha and Kariwo [34] to argue that the once highly respected profession has been completely ‘deprofessionalised’. For children, school attendance is often conditional on both the payment of school fees, and also under-the-table cash ‘incentives’ to underpaid teachers, often beyond the reach of families living in poverty [34].

### Dananai school: our research site

Around 70% of Zimbabweans live in rural areas, and Dananai’s, a pseudonym for the area in which our research school was located, is a typical subsistence farming area. People grow vegetables and other crops, and breed livestock, for their own use, and for sale. People live in homesteads that are either isolated, or clustered in small settlements, such as the one in which our school of interest was located. Here, informal commercial activity involved a handful of small wooden kiosks selling basic supplies and milk, a small clinic as well as a small local church. Children walk to school, sometimes for long distances taking over an hour. The Dananai settlement is located within a larger district comprised of a series of small communities, churches and a mission hospital. Although people are widely dispersed, there is some group activity amongst adults. Many belong to the church, and others belong to community groups such as women’s organisations and savings clubs. There has been sporadic NGO activity to support HIV/AIDS affected people in the area. Chief amongst these have been a local community-based organisation (CBO), which supports orphaned and vulnerable children with food and school related expenses, and the Basic Education Assistance Module (BEAM) a social protection programme spearheaded by the Zimbabwean government and supported by foreign donors that helps children with textbooks, uniforms and school fees.

## Methods

As stated, we draw on a larger multi-method study of school support for HIV-affected children, sponsored by the ESRC-DFID in the United Kingdom. The study received ethical approval from the Medical Research Council of Zimbabwe (MRCZ) and the London School of Economics Research Ethics Committee.

### Data collection

Draw-and-write exercises were carried out in July 2012 with 128 primary school children (58 boys and 70 girls) aged 10–14 in rural Zimbabwe. The draw-and-write approach aims to enable children to articulate their experiences, understandings and feelings through a range of media [35,36]. Three experienced research fieldworkers, all qualified social workers fluent in the local language, were trained in the appropriate research techniques. They invited children from grades 5–7 at a rural primary school in Dananai to participate in the study in a classroom setting. Teachers were not present during the exercise, and all the children who were at school on that day took part in the study. Children participated enthusiastically, viewing the exercise as a break from the usual monotony of a normal school day. As agreed with the Ethics Committees, form teachers gave verbal consent for the participation of the children in their care.

Children were asked to write a story about a child in their school who was affected by HIV, and then to draw a picture to accompany their story. Half the children were asked to write about an HIV-affected boy, and half about an HIV-affected girl. The precise wording of the exercise was as follows: “*How does HIV affect children at your school? Write a story of a child who is affected by HIV (HIV infected/sick relatives/orphaned). What challenges does this child face? How does the school help this child to overcome difficulties in his or her everyday life? Draw a picture to accompany your story.”*

Children had 90 minutes to complete the exercise. As a token of appreciation they were given a school pack containing notebooks, ruler and pens. Children were not asked to disclose whether they were themselves affected by HIV/AIDS. Given the extent of the epidemic in Zimbabwe it is likely that all of them would have had first-hand contact of the impact of the epidemic on peers and family members. The stories were translated by local fieldworkers, closely supervised by the second author. To an extent, the micro-nuances of children’s actual voices are lost through the use of adult translators using terms such as ‘abuse', ‘utensils’ and ‘medication’. However, despite this shortcoming, the stories give a vivid account of children’s views.

### Data analysis

As with our previous experience of draw-and-write in Zimbabwe, the drawings were often unfinished and not particularly clear, with children expressing themselves far more eloquently in the verbal stories [13,37]. For this reason we focus on the stories here. These were analysed using thematic content analysis [38]. We began by coding stories into 45 ‘basic themes’ (descriptive categories reflecting the content of the stories). These were progressively clustered into 17 increasingly abstract and generalised ‘organising themes’ and these into three ‘global themes’. The global themes were: children’s representations of the problems facing HIV-affected school learners; the way these challenges manifested in the school setting; and the impact of schools on children’s coping (see Table 1 for the coding frame, including frequency counts of the mention of themes mentioned across the 128 stories). The global and organising themes form the headings of our presentation of findings below. All names in this paper are pseudonyms to secure anonymity of participants.

**Table 1:** Thematic analysis of children’s stories

**Organising theme**	**Basic theme**	**Issues discussed**	**Freq.**
**Global theme: how challenges of HIV-affected children manifest within the school context**			
HIV-affected households	Household looks visibly poor	Dirty, lack of basic essentials	13%
	Positive perception of household	Beautiful household, clean, livestock	2%
HIV-affected parents	AIDS visible through behaviour of parents	Sleeping, unable to work, visibly sick	6%
Homes of HIV-affected children	Social neglect in household	Isolated, abused, seen as burdens	20%
	Social support in household	Child cared for and happy in household	1%
Responsibilities of HIV-affected children	Caregiving	Bathing, administering medicine, feeding	9%
	Household chores	Fetching water, ploughing fields, cooking	30%
	Chores compromise health & well-being of child	Chores carried out by sick child, chores beyond child’s capability, chores hinder socialisation	27%
	Child engaged in income generation activities	Agricultural work, work for neighbours	2%
**Global theme: how challenges of HIV-affected children manifest within the school context**			
Impact on school attendance	School drop out/late for school	Due to sick parents/sick child, unable to pay fees, chores	15%
Material deprivation	Lack of school equipment	Lack of uniform, books, pens	14%
	Lack of food	Child comes to school without food	20%
	Child looks visibly poor	Torn clothes, Lack of shoes, Dirty	19%
Physical health	Symptoms of poor physical health	Pain, Tired/falling asleep at school, fainting, vomiting	18%
	Child looks visibly sick	Skinny, cracked lips	10%
	Child visits health clinics	Hospital visits	9%
Emotional health	Symptoms of poor emotional health	Cries, sad, miserable	30%
**Global theme: the impact of schools on children’s coping**			
School as a negative context for HIV-affected children	Teacher’s negative response to HIV-affected children	Teacher sends child away from school	2%
		Teacher abuses child	
	Social exclusion	Bullying, lack of friends/Isolation, stigmatisation	30%
Schools as a source of support for HIV-affected children	Bridges between schools and outside sources of support (total 12%)	Referrals to health clinics	3%
		References to support from NGOs, CBOs, BEAM	9%
	Teacher support (total 30%)	Material support: school expenses (fees, books), food/water	24%
		Emotional support: comfort, encourage inclusion, counselling	6%
	Peer support (total 20%)	Material support: Share school resources (books, pens), clothes, food	9%
		Emotional support: Comfort, playing, help with chores	11%
	School as distraction from life tragedies	Learning/playing distraction from problems	5%
	Schools as routes to positive identities	Positive perceptions of children	15%

Given that children were not asked to disclose whether they themselves were HIV affected, and given that they were given the choice of writing a fictional or true story without disclosing which option they chose, to what extent can stories of this nature be seen to reflect the daily lives of our informants? The language and the style of the stories often took a very concrete and specific form (“*There is a boy at our school who is always late*…………” or “*Yesterday in class the girl fainted* …………” or “*At the moment the boy is troubled by his aunt’s death*…………..”). They also often contained degree of detail that suggested that children were referring to specific situations rather than making broad generalisations (e.g. “*if you talk [to the HIV-affected child] about his parents he will start crying but if you talk to him about other things he will respond….”).*

Having said this, we must emphasise that methodologically, the social constructionist framework that informs social representations theory would reject the enterprise of seeking to establish any kind of straightforward one-to-one mapping between stories and the daily ‘realities’ of particular children in our sample. The most we seek to claim is that the representations we identify below constitute powerful symbolic resources which are likely to play a key role in shaping children’s understandings and responses to their everyday life challenges. Our goal is to identify the pool of *collective* symbolic resources available to children, rather than to make claims about the ways in which *individual* children use these representations to inform their individual everyday actions and social relations. The latter enterprise would involve a focus on individual children and a different set of theoretical and methodological starting points. In contrast, social representations are collectively negotiated properties of social groups rather than individuals, and our unit of analysis is the corpus of stories as a whole, and not the accounts of individual children.

The western literature on children’s stories has laid heavy emphasis on the way in which children use stories to provide exaggerated accounts of their fears and anxieties, populating their stories with stock characters such as monsters or wicked stepmothers, more a product of their imaginations than of their daily realities [39]. However, the data in this paper are supported by a large body of research which gives similarly distressing accounts of the problems faced by HIV-affected children in similar southern African settings [7]. The accounts given by the children in our own study are also supported by our larger multi-method study which included surveys, interviews and focus groups with teachers, NGO workers, younger and older children and community members in the same geographical region. Finally a key feature of the western literature is the way in which children’s stories are structured in ways that achieve ‘narrative resolution’ of the problem in focus – the slaying of the monster by a heroic rescuer, or the convenient death of the evil stepmother [40]. Such endings were almost never achieved in our stories.

## Results

### Representations of the life situations of HIV-affected children

#### Lack of supportive adults in HIV-affected households

Parents of HIV-affected peers were depicted as absent, seriously ill, dying or dead.

Sick parents were depicted as visibly sick, sitting down, sleeping on the floor, and as in the following narrative, unable to work:

*He is sad, at their home they have nothing. They they don’t do any work, which shows that they are a family with HIV.* Nyasha, 11.

The illness and absence of parents was reflected in the state of households lacking food and basic essentials, and associated with dead flowers, dying livestock, general dirt and mess:

*This boy bathes a sick person when he comes back from school and there will be no food.* Tatenda, 12.

*Their homestead is messy, they do not sweep and kitchen utensils will be everywhere and they do not clean the house, this makes the child’s life to be miserable.* Rudo, 12.

Lack of caring and supportive adults in the homes of HIV-affected children was a common theme. In some cases parents had passed away leaving children alone as head of households:

*This girl is thirteen. She lives in a small hut. She has no one to live with. Her parents had died two years ago. She wears torn clothes. Her relatives do not love her and she is lonely. And she is homeless. She always took food from a bin. She always cries and thinks about her parents. No one can afford to take care of her.* Yuekai, 13.

In other cases parents were still alive but too ill to take proper care of the child.

*Maria is always facing a lot of challenges like coming to school without eating and not being able to pay school fees. She faces these difficulties because her parents can’t take care of her because they are sick.* James, 12.

#### Children depicted as neglected and abused

Several stories depicted children in HIV-affected households as neglected, isolated and abused:

*This boy is always facing challenges in his life. His parents died because of the pandemic. Nobody looks after the boy.* Vimbai, 14.

*His relatives deserted him saying that they don’t want to take care of someone who is going to die of AIDS.* Rulf, 13.

Children’s vulnerability to abuse dominated many of the stories, some referred to physical abuse:

*No one stays with her because her parents died and she is always crying. Her aunt beat her and she ran away from home and she stays in the forest without food, with the only clothes on her body. This girl is miserable.* Makaita, 12.

Others referred to verbal abuse, usually attached to HIV-related stigma:

*Her grandmother abuses her verbally saying things like “that is why you have HIV”.* Yeukai, 12.

Whilst many stories referred to direct physical and verbal abuse of children, others referred to more indirect abuse in the form of exploitation, discussed below.

#### Household responsibilities of HIV-affected children

The involvement of children in household duties is a widely accepted aspect of children’s socialisation in southern Africa [41,42]. Our stories often suggested HIV-affected children in this context were carrying an unreasonable burden of responsibilities. They were often depicted as doing this in the absence of adequate food to sustain their strength, with chores often leaving them exhausted and hungry, and sometimes causing them to be late for school or to miss it altogether. Most common were fetching water and firewood, caring for livestock, cleaning the house, cooking and washing clothes and dishes. A small minority referred to children engaging in income generating activities. Several spoke of children’s engagement in intimate caregiving activities for sick parents – most often the bathing of sick parents, and less often dispensing medicine:

*Before he comes to school he makes sure that he has fed them, given them their medication and they have all that they need for the day.* Lindiwe, 14.

Often these duties were depicted as exploitative, compromising the needs, safety and well-being of their HIV-affected peers. Children with sick relatives tended to have the heaviest responsibilities including nursing care and heavy household chores:

*She wakes up and does everything for her parents. She cooks for them, fetches water and does all the chores at home. When she finishes the chores she then goes to school. Every day she is the last person to get to school.* Tapiwa, 14.

*This boy is a very troubled boy before he comes to school he cleans the house, does the dishes and fetches water for the household. This boy does these chores very early in the morning and when he is about to leave for school he is told to first prepare food and also boil bathing water by the mother.* Albert, 12.

Chores at night were also depicted as a challenge:

*At home he is abused by the grandmother. He goes to herd cattle without having eaten any food and when he comes back it will be already night and he sleeps without eating.* Tarisai, 12.

Stories also depicted relatives as exploiting orphans or foster children in their care. Some stories spoke of children carrying out significantly heavier chores than other household members, which hindered their possibilities for social engagement and playing with others:

*When he gets back from school there will be no food prepared for him and there will be chores for him. He does all the work at home while his relatives are basking in the sun.* Edith, 12.

*This girl now stays with her father’s young brother and the people who she stays with are always abusing her. Her life is all about suffering. She does the household chores by herself while the other children are playing.* Constance, 14.

Several stories spoke of HIV infected children in pain or visibly sick, with chores beyond their capability and strength:

*This boy is sick. He goes to the hospital but he does work all the time though he is sick. The boy stays with his grandmother and grandfather. He fetches water and many other chores.* Peter, 11.

*The boy l know of is always sick. He is sent on errands that he cannot do like fetching water or to carry things that he is unable to carry.* Mazvita, 12.

In short, responsibilities of HIV-affected children were represented as distinctively challenging, and at times depicted as abuse by relatives when: i) the child carried out significantly heavier responsibilities than the rest of the household, especially at night, ii) when responsibilities compromised the child’s opportunities for positive social engagements with peers, and access to education and iii) when responsibilities were carried out by undernourished and sick children.

### Representations of how these problems manifested in the school context

#### Impact on school attendance

One of the most common observations was how affected children’s home situations impacted on school attendance, through illness, nursing obligations or inability to pay school fees:

*These days she is no longer coming to school due to her illness.* Shelter, 12.

A*t times she comes late to school as she first bathes and clothes her parents.* Ralph, 13.

*She doesn’t go to school because she had no money to send herself to school.* Yuekai, 13.

#### Impact of material deprivation on schooling

HIV-affected learners were often depicted as poorer than other children. Their poverty impacted on their physical appearance: visibly poor, dirty, poorly dressed in rags, torn clothes and uniform:

*He is given dirty and torn clothes to wear. No one washes the clothes for him so they are always dirty.* Mazvita, 12.

Some stories referred to children lacking basic school equipment such as pens, books, and notebooks in the classroom:

*She doesn't pay fees for her. There are people who pay fees for her and when she comes to school she doesn’t have books.* Morgan, 13

More commonly stories spoke of children coming to school without adequate food and water, with one additionally highlighting the negative consequences of taking antiretroviral treatment without adequate food:

*…the only thing that pains me about her is that she does not carry food to school even though she takes pills. I know that someone who has HIV should eat healthy food since she will be taking pills.* Faith, 12

#### Poor physical health

When talking about children in school settings, references were often made to them showing physical symptoms of severe illness and visiting health clinics during school hours. Some stories referred to children vomiting and fainting in school, and noted how the physical condition of HIV-affected peers impacted on their daily school lives in comparison to other children, sometimes influencing children’s ability to concentrate in class:

*At school she would get outside the classroom and we would see her vomiting.* Gary, 12.

*The boy suffers from HIV and AIDS… When he is at school he won’t be concentrating like other children.* Florence, 14.

Other spoke of how physical pain, hunger and sleepiness challenged children’s ability to walk to school or participate fully in school activities, either due to their HIV infection or lack of adequate care:

*At times she would come while she was sick and she would just sit. At school she would face a lot of difficulties, she would at times faint with hunger or she wouldn’t have a pen… She was always saying that she was feeling cold.* John, 12.

*This boy takes care of sick people at his home. Their home is dirty because they do not have time to clean the home. He faces difficulties at school He walks slowly when he is going to school because he will be in pain.* Cynthia, 12.

#### Poor emotional health

HIV-affected children were frequently depicted as emotionally distressed, crying in the playground, brooding over worries about their home situations and living in fear of finding their parents dead at home in the evening:

*When we are going to school if you talk about parents he will start crying but if you talk about other things he will respond.* Para, 13.

*The aunt died from AIDS and when he thinks about it he starts crying. At the moment he is troubled by it. On the day that the aunt died he cried. If you start talking about it he still cries.* Kundai, 13.

Most narratives concerning emotional distress were linked directly to some aspect of the child’s predicament:

*Her illness makes her miserable.* Patience, 13.

*This boy is always crying because of his sick parents.* Cecilia, 12.

*She is constantly stressed due to the fact that she is an orphan.* Judith, 12.

*He is always crying as he is abused by the people he stays with,* Taremekedzwa. 10.

Furthermore, children frequently commented on the impacts of emotional distress on the behaviour of HIV-affected peers:

*He goes to school but he doesn’t do anything. He doesn’t even read he just sleeps because he will be unhappy.* Joseph, 12.

*At school she was so lonely and no one got near her saying if your parents have HIV you have it too. Sometimes she spends most of her time in tears,* George, 12.

### The impact of schools on children’s coping

#### Representations of negative impacts of schools on children’s coping

Whilst the aims of our study were to explore the role of schools in supporting children, some stories suggested schools sometimes served as a negative space for HIV-affected children. References to bullying (including stigma-related bullying) were frequent:

*She provided care for her parents alone and other children used to laugh at her about her parents’ illness… When she was at school other children laughed at her and were bullying her all the time… She used to go to school without shoes. Other children looked down upon her.’* Elisabeth, 12.

*When people knew the problem they started laughing at her. At school she was so lonely and no one got near her saying if your parents have HIV you have it too. Sometimes she spends most of her time in tears.* George, 12.

*John can no longer play with other children because they say he suffers from HIV.* Dancan, 12.

Despite frequent references to bullying and social exclusion, nearly as many stories expressed sympathy for HIV-affected peers

*I feel sorry for Maria because the other girls don’t want to play with her because they say that she has HIV.* James, 12.

*You will see the child crying and you feel pity.* Rudo, 12.

#### Schools as useful bridges between children and outside sources of support

A few participants referred to links between schools and external sources of support for their HIV-affected peers. Most referred to children receiving support from local CBOs and NGOs who assisted with payment of school fees. A handful of references were made to schools facilitating children’s access to health services:

*At school an organization called XX (a local CBO) helps to pay his school fees.* Mercy, 11.

*The school head said she would take the girl to the hospital every month to get the pills she needed.* Patience, 13.

### Provision of material and emotional support by teachers

Some stories referred to teacher initiatives to support children. Most frequent reference was made to teachers providing help with school related expenses such as books, pens and uniforms:

*The teachers are the ones who gave her [money] so that she would go to school like what the others are doing,* Anengoni, 15.

Reference was also made to teachers helping with food, water and clothing:

*At school the school head was sympathetic to her and started giving her food and clothing,* Elisabeth, 12.

One reference was made to a headmaster helping a sick child with her household duties by helping her fetch water for her household.

*The school head assists her by carrying her in his car. He goes every morning to fetch her in his car. Her parents do not work they stay at home. The headmaster pays school fees for her. When it is time to eat she eats at the teacher’s house. If she does not have a pen she is given by the teacher,* Bastirai, 13.

And in another story, the head master recognised the poor physical condition of the child, referring her to hospital and assisting with food:

*The school head once talked to her and she said she always faints when she is walking and she vomits when she is in class. The school head told her that she will be given food at school, and took her to the hospital.* Patience, 13.

Only very few stories referred to situations where a teacher had responded to the emotional needs of the children:

*Sometimes teachers take him when he is crying.* Para, 13.

Another story referred to teachers offering guidance and advice to a child:

*At school teachers help her to follow rules and be on time and other things. She faces so many challenges so much that even the teachers sympathize with her.* Holley, 13.

Finally, a few stories recognised the potential for teachers to respond to HIV-related bullying:

*The teachers noticed that she wasn’t concentrating and then they told her to stay at the school and that if anyone was to speak badly to her they would be called to the office or given a punishment. Olivia is now living happily because no one laughs at her and this makes me happy.* Yundai, 12.

### Provision of material and emotional support by peers

Some stories referred to children sharing food and school materials with HIV-affected peers:

*When he is at school other school children help him. He does not eat anything before coming to school so other school children give him food,* Victoria. 14.

Other stories referred to learners helping HIV-affected peers with heavy household duties:

*She is told to go and fetch water at night. If we see her we give her the water because it will be too dark. We once went to stop her from being beaten by her aunt because she came home late looking for cattle,* Chipo, 12.

A few stories referred to peers providing comfort and support through play and interaction:

*Tapiwa was always crying when he came to school and we would comfort him and he would stop crying. We would play different games with him.* Andy, 14.

In a few stories, participants described situations where children had served as advocates for their HIV-affected peers:

*At school other children used to laugh at her because her parents had died of HIV. We discussed this issue with the teachers and told them about this story so now the teachers are helping such children by giving them pens. These children are no longer unhappy because all those who used to laugh at the children are now being beaten.* Jean, 13.

*Her friends are the ones who reported the issue to the school head. They talked to the girl and told her that if she had told them earlier she would have got early treatment before the illness was serious.* Yekai, 11.

Whilst references to schools as sources of support were relatively few and far between across the data set, there were a few stories that referred to both teachers and peers helping HIV-affected children in various ways.

### School as distraction from life tragedies

Several participants depicted learning as a source of distraction and represented the school environment as providing opportunities for HIV-affected peers to be happy, play freely and forget about their problems.

*What makes him happy when he is at school is when he plays with the others and tells them about the challenges that he face. He forgets his problems because he will be playing with the others.* Michael, 12.

*When he comes to school he is happy because it makes him forget that his parents are dead all he will be thinking of is to learn.* Paul, 12.

A few narratives made a clear distinction that associated home with negative emotions and school with positive emotions:

*This boy has many difficulties so when he is at school he will be happy but when he gets home he becomes sad.* Victoria, 14.

### School attendance providing opportunities for construction of positive identities by HIV-affected children

Whereas most stories emphasised vulnerability and weakness in the face of the severity of their life challenges, some included positive moral evaluations of HIV-affected peers. These emphasised their strength of character and moral purpose with adjectives such intelligent, talented, responsible, obedient, hard working and good in school. Whilst the following story represents the child as emotionally troubled and materially deprived, she is not simply depicted as a victim, with the story-teller emphasising her positive traits.

*She comes to school without food always so her friends give her food. Her clothes are torn. Sometimes she won’t be writing and if you ask her why she will say she does not have a pen. She cries a lot. But she is intelligent is always at the top of the class. Many teachers like her.* Lavenda, 11.

Another participant spoke of how successful school performance had a positive impact on the emotional well-being of the child in his story:

*When he comes to school he is happy because he is good in school.* Tonderai, 10.

Most positive descriptions were associated with school performance, but a handful referred to other skills and accomplishments such as sporting prowess or agricultural skills:

*Tifus was especially good in soccer and no one would play better than him.* Andy,14.

*This boy is good in farming maize and vegetables. He produces maize to sell so that he can buy food.* Gabriel, 12.

## Discussion

How do children in Sub-Saharan Africa understand how HIV/AIDS impacts on themselves and their peers? How do they view the school-related resources available to them for coping, often in the absence of significant support from adult carers? As stated above, much previous research has looked at the interaction of schools with external NGOs and interventions seeking to support HIV-affected children. With the retreat of development funding, however, policy makers are increasingly looking at what can be done by schools in the absence of outside support and intervention. What do our findings highlight about sources of already existing indigenous ‘best practice’ to build on in this regard?

Our findings have shown how children’s stories gave often quite harrowing accounts of their HIV-affected peers in daily battle with often physical, emotional and social challenges. We turn briefly to our counts of the frequency of themes across the 128 stories (see Table 1) as a way of pulling together our findings, with a particular interest in emphasising (i) the heavy overall emphasis of children’s stories on the *emotional* impacts of HIV on affected children, and the limited support provided by school-based relationships and networks in coping with these, and (ii) the way in which fear and stigma seemed to limit supportive peer responses to HIV-affected children, despite a strong degree of peer recognition of their suffering and a degree of implicit or explicit peer respect for their efforts to cope with adversity.

Our first global theme categorised children’s representations of the life situations of HIV-affected children. Across the corpus of stories, heaviest emphasis was put on their engagement in household chores to a degree regarded by their peers as unreasonable and as excessive (a theme in 30% of the stories), and to an extent which was said to compromise their well-being (27%). Reference was also made to their ‘social neglect’ understood as experience of isolation and abuse in households and being made to feel they were a burden (20%).

Our second global theme categorised representations of how children’s life problems impacted on their experiences at school. Here emphasis was most frequently placed on their emotional distress (30% of stories depicting children as sad or crying) and their physical ill-health represented by symptoms such as pain, exhaustion, vomiting and fainting (18%). There was also reference to social impacts including coming to school without having eaten (20%), arriving late at school (5%), a problem general regarded extremely negatively by teachers in this setting, and lacking essential school equipment, such as uniforms, books and pens (14%).

Our third global theme, documenting children’s depictions of the impact of schools on children’s coping with these issues, links most directly to our interests in schools as a potential source of health-enabling social capital. Here we remember that participation in social relationships can have negative as well as positive impacts on health and well-being. In the stories, the space children devoted to accounts of *challenges* facing children in the school setting far exceeded the space devoted to sources of school-related *support*. Thus, for example, one third of the stories depicted school as a place characterised by the social isolation, bullying and HIV-stigma, reinforcing the sense of how school relationships reinforced their wider experiences of rejection, loneliness and desolation. Nevertheless, even against the background of this unpromising account of the school setting, some children depicted schools as presenting their HIV-affected peers with the possibility of a degree of support from four sources: (i) teachers (a total of 30% of the stories referred to one or more forms of support from a teacher); (ii) peers (a total of 20% of the stories referred to one or more forms of support from a peer); (iii) distraction from their daily suffering (5%); and (iv) a sense of positive identity (15%). Schools also served as a point of access to assistance from NGOs and other agencies (esp those providing school fees) (12%).

Teachers were most often depicted as offering children sources of material support, in the form of books, pens, referrals for fee assistance and food or water (24%) and referrals of children to health clinics (3%). In the face of the frequency of children’s references to the emotional suffering of their HIV-affected peers, however, references to teachers comforting distressed children were relatively few (6%). This resonates with our companion study of rural Zimbabwean teacher’s accounts of their ‘ethic of care’ towards HIV-affected children [Campbell C, Andersen L, Exploring the ethic of care among rural Zimbabwean teachers, submitted.] Here, in interviews with teachers, despite making frequent references to the potential for teachers to provide such children with emotional support, teachers acknowledged that in practice they seldom responded directly to children’s emotional distress, and were more likely to provide support of the material kind. In our children’s stories, peers were slightly more likely to offer emotional support in the form of comfort, play and help with home chores (11%) in addition to material support (9%). However this potential emotional support was offset by frequent references to bullying and stigma (30%).

Overall the stories suggest that the types of supportive relationships that were available to children – mainly with teachers and peers – were limited in the face of the enormity of the challenges they were facing. Furthermore their support tended to be of the practical or material sort. Relatively little of the support that was seen to be available from teachers and peers tackled what children depicted as the main problem in their stories: the emotional distress, fear and isolation arising from their life stresses. In this regard our research participants were all very clearly attuned to the emotional suffering of their peers. Nearly every single story contained a reference to at least one type of emotionally distressing experience suffered by the protagonist, be it bullying, exhaustion, fear, abuse by relatives and so on.

In a sense, our child authors gave complex and nuanced insights into the plight of their peers. Whilst acknowledging the extent of their personal tragedies, they were seldom portrayed as complete victims, many stories acknowledged their strengths as well as their vulnerabilities. Furthermore, stories were often overlaid by an intangible and implicit sense of the moral frames of the children, both authors and their protagonists. These were centred around a sense of common respect for and commitment to the value of hard work, an implicit celebration of the value of caring and loyalty to one’s parents and family members, the importance of contributing to the reproduction of one’s household and its members, the importance of respecting the value of school education and so on. These were also sometimes expressed explicitly, as in the references to HIV-affected children as obedient, intelligent, hard-working and good-hearted (15%) and in stories they spoke of how the fact that they were managing to attend school served as a comfort and distraction from their daily hardships (5%).

## Conclusion

In summary, we argue that our findings present a less than optimistic picture of the potential for schools to serve as an ‘indigenous’ source of support for HIV-affected children. The extent to which children regarded schools as a source of sustaining and health-enabling social relationships was ambiguous and contradictory, challenging policy assumptions that schools are intrinsically well-placed to ‘substitute for families’ and to support children in responding to the extraordinarily complex and multi-faceted physical, emotional and social challenges. Care needs to be taken in assuming that schools are in some sense ‘separate’ from surrounding communities in an un-problematized way that positions them as an obvious location of solutions to community problems – with problems located outside of the school - as if school and community could be conceptualised as separate spaces. Of course, schools are the institutional setting that children regularly engage with most regularly, and teachers would seem to be a key support resource in principle. Yet our findings suggest that rather than children regarding schools as a haven for HIV-affected peers from the hardship, isolation, rejection and stigma that faced them in their households and neighbourhoods, the school was in some ways very much a part of these communities and an extension of these negative relationships. Our stories did not give much indication of schools or teachers serving as sources of indigenous and bottom up best practice (what development agencies might call ‘citizens doing it for themselves’ in tackling problems without much outside help) given the enormity of children’s problems and the tremendous constraints teachers are operating under.

It may be the case that with (i) significant external support, resources and training; and (ii) and a promotions and reward system that recognised and rewarded the additional “emotional labour” [43] and time commitment involved in offering pastoral care and social protection to very vulnerable children, that schools and teachers could indeed be assisted to make a greater contribution to the challenges of offering care and protection to the children in their classes [Campbell, C, Andersen, L. Exploring the ethic of care among rural Zimbabwean teachers. Submitted]. Presently the development of a wide range of materials and initiatives to develop such support systems for teachers is in process in Zimbabwe and South Africa. These include the recent development of a Diploma for Teachers’ Diploma on Care, Support and Protection [44] and the development of ‘Caring Schools’ policies in South Africa [45-47]. Furthermore, all Zimbabwe schools are required to have an AIDS policy [48,49] although in the current financial and political crisis, many rural schools are battling to carry out their traditional mandate of book learning, let alone more complex new caring functions. As a result, such policies may often only been rolled out in partial ways, or not at all, in some contexts. Teachers in our wider multi-method study reported feeling a complete lack of formal institutional support for the provision of care to HIV-affected learners. There is a need for on-going efforts to ensure that additional capacity and resources are directed at developing and actioning such policies more widely in Zimbabwe, particularly in rural schools.

However a large literature on the care and support of vulnerable groups in Northern countries suggests that care is not a quality that can be conjured up by policies and regulations in the absence of parallel efforts to create “emotional environments” that support and enable caring relationships [43,50]. Research with Zimbabwean teachers suggests that they often feel underpaid and demoralised; many of them with HIV themselves or in a state of continual fear at the vulnerability of themselves and family members [51,52], in institutional settings where they feel that too little formal recognition is given to the value of non-academic support for children by school principals whose attention is often fully preoccupied with fulfilling their traditional mandates of book learning.

In relation to our research methodology, namely eliciting children’s own views of their HIV-affected peers, some might argue that children are not necessarily consciously aware of the support structures that are put into place for them, and that it may have been the case that more was being done for HIV-affected children by schools than our child authors acknowledged. Others would argue that children’s own accounts of their daily lives need to be taken seriously, and caution against a model of children as less qualified to comment on their own experiences than others. We would cautiously side with the latter position on this debate, particularly given that these children’s accounts were consistent with accounts given in interviews and focus groups with teachers and community members in other sub-investigations in our larger study as well as more general studies of the general difficulties faced by HIV-affected children in Zimbabwe [7] and accounts of the more general challenges that the Zimbabwean economic and political situation has placed on the teaching profession [34]. Furthermore, in response to those who argue that children in our study may have been located in an atypically unhelpful school, extensive M + E and survey data (associated with our wider study) placed our case study school as relatively successful in ensuring the attendance and health of its HIV-affected pupils compared to other schools in the region, so the study school for this particular paper cannot be regarded as an unduly negative case [Pufall E, Gregson S, Eaton J, The contribution of schools to supporting the well-being of children affected by HIV in eastern Zimbabwe, submitted].

In conclusion, drawing on children’s own accounts of their peers’ experiences, in a framework which respects the ability of children to give valuable accounts of their social worlds, our paper suggests that the situation of many HIV-affected children in Zimbabwe continues to be extremely negative. The policy assumption that teachers and schools can be regarded as an already existing indigenous resource capable of filling the gap left by absent or chronically ill parents or relatives in the absence of a very significant increase in outside training, support and additional resources (from e.g. public sector, or international donors), may be an extremely over-optimistic one. Assumptions that schools have the potential to carry this role should not be allowed to serve as a smokescreen for the extent of children’s suffering, or the lack of support available to them in some settings.

## Competing interests

The authors declare that they have no competing interests.

## Authors’ contributions

The overall study was designed by CC and MS. CC wrote the manuscript, incorporating a data analysis section that was drafted by LA. Each author made a significant contribution to the intellectual content of the paper, through regular meetings (i) to plan and execute the data collection, (ii) to discuss interpretation of data; and (iii) through on-going discussions of the intellectual content and conclusions of the paper. All authors read and approved the final manuscript.

## Pre-publication history

The pre-publication history for this paper can be accessed here:

http://www.biomedcentral.com/1471-2458/14/402/prepub
